# Progressive Feeding Intolerance Secondary to a Congenital Spinal Teratoma in a Four-week-old Female

**DOI:** 10.7759/cureus.3281

**Published:** 2018-09-11

**Authors:** Shane C Rainey, Bhavana S Kandikattu

**Affiliations:** 1 Pediatrics, University of Illinois College of Medicine At Peoria, Peoria, USA

**Keywords:** teratoma, pediatrics, oncology, hospital medicine, teaching hospital, neurosurgery

## Abstract

A poorly feeding neonate presents the clinician with a diagnostic challenge. Feeding difficulties and irritability may be due to sepsis, congenital heart disease, inborn errors of metabolism, non-accidental head trauma, as well as a vast variety of other pathologies. Teratomas are rare pediatric tumors that can occasionally present in the immediate neonatal period and can manifest in the infant's central nervous system (CNS) with non-specific symptoms of poor feeding, lethargy, and somnolence. Operative resection remains the cornerstone of treatment; however, there is no well-defined role for adjuvant chemotherapy or radiation in these treatments. We report a case of a four-week-old female presenting with progressive feeding intolerance secondary to a near holocord thoracic spinal teratoma. Her tumor was surgically resected and she was treated with adjuvant chemotherapy and radiation for 13 months and is now in clinical remission. While rare, intramedullary spinal cord lesions should be considered in the differential diagnosis of infants presenting with poor feeding and hypotonia.

## Introduction

A poorly feeding neonate presents the clinician with a diagnostic challenge. Frequently, feeding difficulties and irritability are attributed to sepsis, a common presentation in the immediate neonatal period that can present without other classic signs such as fever or temperature instability [1,2]. However, the differential diagnosis of a poorly feeding and irritable neonate is broad and includes other conditions such as congenital heart disease, inborn errors of metabolism, non-accidental head trauma, neurological disease, as well as a vast variety of other pathologies [2,3]. We present a case of a neonate with poor feeding secondary to a space occupying spinal cord tumor.

## Case presentation

A four-week-old female born at term via unremarkable spontaneous vaginal delivery presented with a one-week history of irritability, poor feeding, and progressive somnolence. Prior to the onset of symptoms, her newborn period was unremarkable with good appetite, growth, voiding, stooling, and weight gain. Per parental report, she had a normal neurological exam in the nursery and at her newborn and two-week well child evaluations. She then began to have progressive feeding difficulty, becoming very irritable with feeds. She also became irritable with any attempted movement of her upper extremities. There were no fevers or hypothermia noted at home. Family and social histories were noncontributory.

**Figure 1:**
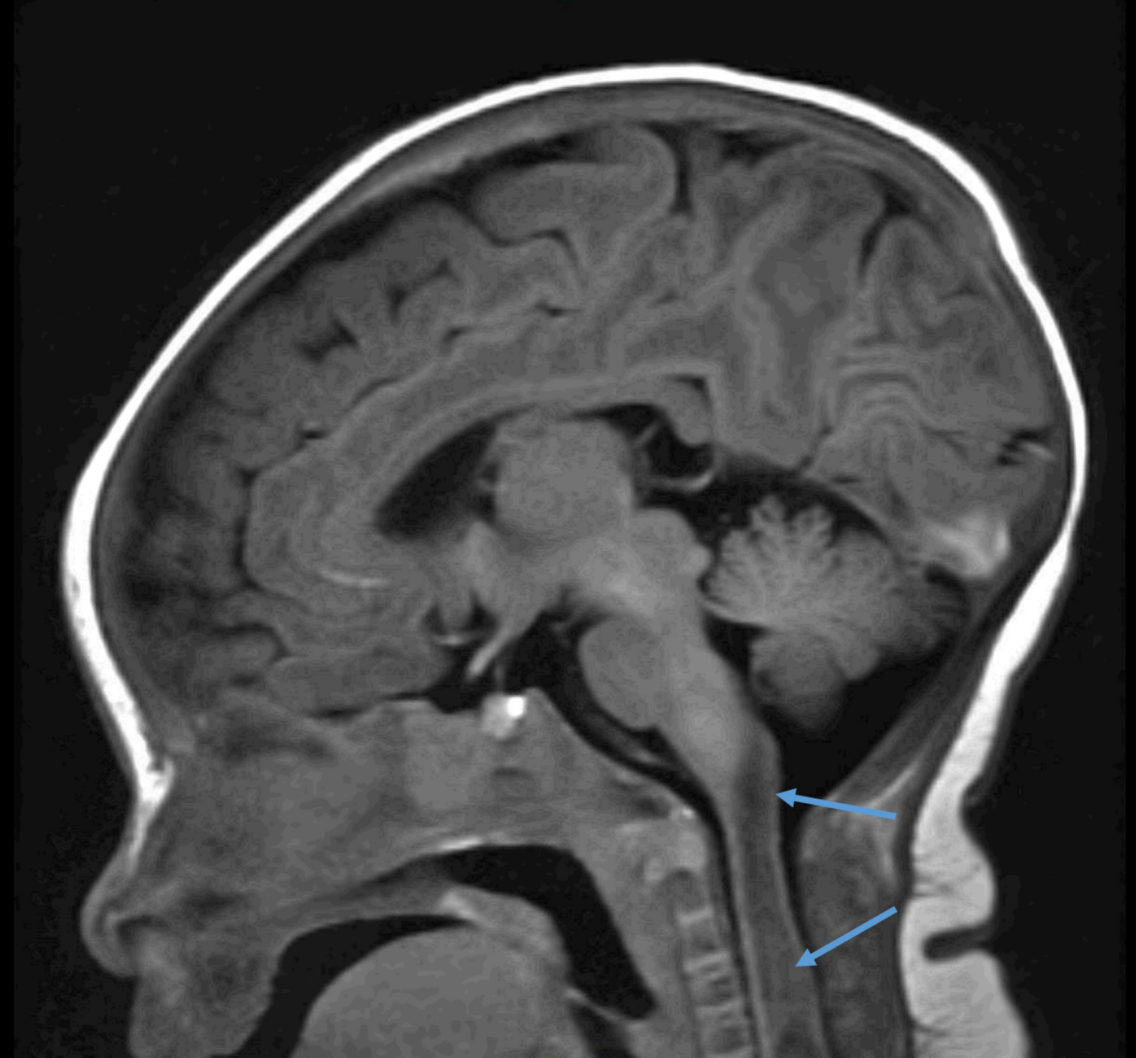
MRI Brain. MRI of the brain demonstrating abnormal signal enhancement in the proximal cervical spinal cord. MRI: Magnetic resonance imaging

**Figure 2:**
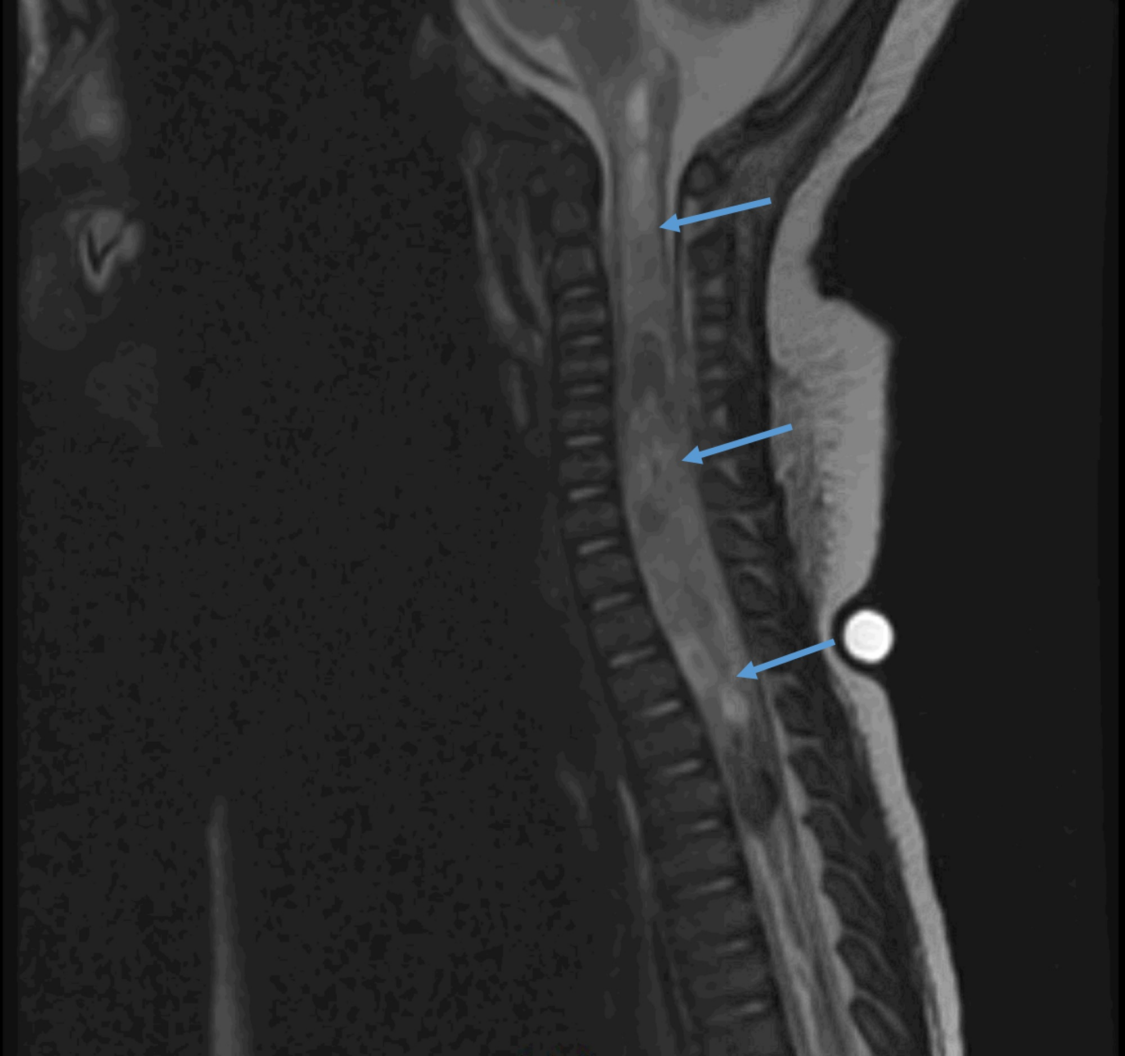
MRI Spine. MRI of cervical, thoracic, and lumbar spine demonstrating a near holocord hemorrhagic, intramedullary mass consistent with a congenital immature teratoma. MRI: Magnetic resonance imaging

On physical examination, she was afebrile with a heart rate of 130 beats/minute, respiratory rate of 40 breaths/minute, and irritable with any attempts at examination. Her head was normocephalic and her fontanel was soft and non-bulging. Her cardiac exam was without murmurs, her lungs were clear bilaterally, and her capillary refill was less than two seconds. Neurological examination was notable for absent bilateral Moro reflexes and decreased bilateral upper extremity grasp reflexes. Emergent computed tomography of her head was negative for an acute intracranial process. She was admitted to the inpatient ward where a lumbar puncture yielded slow-flowing, grossly xanthochromic fluid containing 132 nucleated cells with a normal differential. Ampicillin and cefotaxime were started. Blood, urine, and spinal fluid cultures were negative. She remained irritable and, over the next 12 hours, developed progressive hypotonia and areflexia of her bilateral lower extremities. Magnetic resonance imaging (MRI) of her brain was subsequently performed, which was also negative for acute intracranial pathology but demonstrated signal enhancement in the proximal cervical spinal cord (Figure 1). Due to this finding, further imaging with cervical, thoracic, and lumbar MRI was completed, revealing a near holocord hemorrhagic, intramedullary mass (Figure 2). Neurosurgery and oncology were urgently consulted and the patient was taken to the operating room for surgical resection on hospital day three. Pathology confirmed the diagnosis to be a congenital immature teratoma.

She was electively treated with carboplatin, etoposide, and ifosfamide chemotherapy along with radiation after surgical resection based on the Children's Oncology Group ACNS 1123 protocol. She completed this regimen after approximately 13 months and is currently in clinical remission. She has gross developmental delays; however, her motor function and residual neurological deficits are slowly improving with the help of early intervention.

## Discussion

Central nervous system (CNS) teratomas are exceedingly rare. Teratomas make up approximately 3% of all pediatric tumors and 0.1% of all CNS tumors [4,5]. Of these CNS teratomas, roughly 0.2–0.5% occur in the spinal cord [5]. Teratomas are cystic or solid tumors that often consist of elements from all three germ layers and are most commonly found in the ovary in adult females and in the sacrococcygeal region in children [4]. Depending on the amount of tissue differentiation, they are classified as either immature, mature, or malignant [6]. Mature teratomas contain tissues that are appropriately developed, including bone, hair, teeth, nerve, cartilage, and muscle while immature teratomas contain more primordial tissues such as neuroepithelial tissue [4]. Malignant teratomas may consist of elements of both immature and mature subtypes, but also contain fragments of other germ cell tumors such as yolk sac tumors or choriocarcinoma [4]. The diagnosis is made histologically either via biopsy or at the time of surgical resection. Operative tumor removal remains the primary therapy and gross total resection offers the possibility of cure [4]. There are case reports of adding adjuvant chemotherapy and radiation in the case of subtotal resection, although no standard of care exists for these treatments [7]. In relation to our present study, we employed both total surgical removal as well as adjuvant chemotherapy to rid the patient of any residual tumor that might be present to enable remission of her CNS teratoma. The relative paucity of literature renders the prognosis of these tumors difficult to estimate, thus many patients with immature intramedullary teratomas have died within one year following surgery [8].

## Conclusions

Intramedullary spinal cord lesions should be considered in the differential diagnosis of infants presenting with poor feeding, irritability, and hypotonia. A high index of suspicion is necessary for their diagnosis. Given that focal neurological findings can be subtle in infants, a thorough initial and repeated neurological examination should be a cornerstone of physician evaluation.
